# Effect of Ketamine/Propofol Admixture on Peri-Induction Hemodynamics: A Systematic Review and Meta-Analysis

**DOI:** 10.1155/2020/9637412

**Published:** 2020-05-08

**Authors:** Nathan J. Smischney, Mohamed O. Seisa, Allison S. Morrow, Oscar J. Ponce, Zhen Wang, Muayad Alzuabi, Katherine J. Heise, Mohammad H. Murad

**Affiliations:** ^1^Department of Anesthesiology and Perioperative Medicine, Mayo Clinic, 200 First St. SW, Rochester, MN 55905, USA; ^2^HEModynamic and AIRway Management Group, Mayo Clinic, 200 First St. SW, Rochester, MN 55905, USA; ^3^Evidence-Based Practice Center, Mayo Clinic Robert D. and Patricia E. Kern Center for the Science of Health Care Delivery, Mayo Clinic, 200 First St. SW, Rochester, MN 55905, USA; ^4^Universidad Peruana Cayetano Heredia, Av. Honorio Delgado 430, San Martin de Porres 15102, Lima, Peru; ^5^Division of Preventive, Occupational, and Aerospace Medicine, Mayo Clinic, 200 First St. SW, Rochester, MN 55905, USA

## Abstract

To evaluate the effectiveness of an admixture of ketamine and propofol on peri-induction hemodynamics during airway manipulation, we searched electronic databases of randomized controlled trials from January 1, 2000, to October 17, 2018. Trial screening, selection, and data extraction were done independently by two reviewers with outcomes pooled across included trials using the random-effects model. We included 10 randomized trials (722 patients, mean age of 53.99 years, 39.96% female). American Society of Anesthesiologists physical status was reported in 9 trials with classes I and II representing the majority. Ketamine/propofol admixture was associated with a nonsignificant increase in heart rate (weighted mean difference, 3.36 beats per minute (95% CI, −0.88, 7.60), *I*^2^ = 88.6%), a statistically significant increase in systolic blood pressure (weighted mean difference, 9.67 mmHg (95% CI, 1.48, 17.86), *I*^2^ = 87.2%), a nonsignificant increase in diastolic blood pressure (weighted mean difference, 2.18 mmHg (95% CI, −2.82, 7.19), *I*^2^ = 73.1%), and a nonsignificant increase in mean arterial pressure (weighted mean difference, 3.28 mmHg (95% CI, −0.94, 7.49), *I*^2^ = 69.9%) compared to other agents. The risk of bias was high and the certainty of evidence was low. In conclusion, among patients undergoing airway manipulation and needing sedation, the use of a ketamine/propofol admixture may be associated with better hemodynamics compared to nonketamine/propofol sedation. This trial is registered with CRD42019125725.

## 1. Introduction

Peri-intubation hypotension, defined by either systolic blood pressure or mean arterial pressure (MAP) below a certain threshold (i.e., <90 mmHg or <65 mmHg) or the introduction of vasopressors, has been recognized as a potential target area for research given its association with patient-centered outcomes. For example, peri-intubation hypotension has been associated with both increased length of stay and mortality [1–3]. Furthermore, this association has been identified not only in the critically ill but also in elective surgical patients [4, 5]. Several studies indicate that the frequency with which peri-intubation hypotension occurs is fairly high, with one report indicating an incidence of greater than 80% [1, 2, 5, 6]. However, the data presented on the frequency is dependent on definitions currently used in the literature for which no standard consensus exists. Perhaps, the best evidence for peri-intubation hypotension incidence in the critically ill comes from Green and colleagues [7]. They performed a systematic review of emergent intubations performed outside the operating room and found that the incidence of peri-intubation hypotension ranged from 5 to 440 cases per 1,000 intubations (0.5–44%) with a pooled estimate of 110 cases per 1,000 intubations (11%) [7]. Thus, peri-intubation hypotension is not uncommon, and given the associations observed in the literature, prevention of peri-intubation hypotension is likely to improve patient-centered outcomes.

Several putative risk factors have been implicated in the pathway to peri-intubation hypotension. Age, illness severity, and preintubation hemodynamic derangement have consistently been implicated in the development of peri-intubation hypotension [2, 3, 8, 9]. One modifiable risk factor potentially leading to peri-intubation hypotension is the choice of intravenous anesthetics with some anesthetics (i.e., propofol and barbiturates) more likely to lead to peri-intubation hypotension than others (i.e., etomidate and ketamine) [10]. Lately, a novel intravenous anesthetic admixture has gained popularity based on potential hemodynamic preservation postadministration [11–13]. The admixture involves the combination of propofol with its vasodilatory effects balanced by the vasoconstricting properties of ketamine [10].

The majority of studies on ketamine/propofol admixture have evaluated critically ill patients in the emergency department with the evidence demonstrating a potential sparing effect on hemodynamics along with improved pain relief and sedation quality. These studies have evaluated ketamine/propofol admixture from the standpoint of a continuous infusion for procedural sedation and analgesia [14–16]. There have been a couple of systematic reviews on ketamine/propofol admixture sedation, demonstrating that ketamine/propofol admixture appears safe and efficacious for procedural sedation and analgesia and is possibly better than propofol only at reducing cardiorespiratory problems [17, 18]. The wealth of the evidence above has mainly focused on ketamine/propofol admixture use in terms of infusions for procedural sedation and analgesia. There are limited studies addressing the potential hemodynamic preservation effects of the admixture when administered as an induction agent for endotracheal intubation. Given the above associations between peri-intubation hypotension and increased patient morbidity and mortality, and the mounting evidence with ketamine/propofol admixture as an agent that allows potential maintenance of hemodynamics when administered for endotracheal intubation, our aim was to perform a systematic review and meta-analysis on the hemodynamic effects of ketamine/propofol admixture when administered as an induction agent for airway manipulation such as endotracheal intubation.

## 2. Methods

We used the Preferred Reporting Items for Systematic Reviews and Meta-analyses (PRISMA) 2015 statement to report the trial [19–22]. All reviews were conducted by two independent reviewers (MS and AM). Data collection was performed from January 1, 2000, to October 17, 2018. A formal protocol does not exist for this systematic review and meta-analysis. The review was registered with the International Prospective Register of Systematic Reviews (CRD42019125725).

### 2.1. Eligibility Criteria

Randomized controlled trials published in peer-reviewed journals and written in English were eligible if they included the following: (1) adult patients who received ketamine/propofol admixture as an induction agent in procedural areas and underwent airway manipulation such as endotracheal intubation or supraglottic device placement; (2) a comparison to other induction agents including propofol, ketamine, etomidate, and sodium thiopental or any other combination; and (3) a report of hemodynamic effects (heart rate, systolic and diastolic blood pressure, and MAP) during the first 10 minutes after the induction of general anesthesia in American Society of Anesthesiologists I II, III, and IV patients. The intervention must have equal or similar doses (e.g., ketamine/propofol admixture 1 : 1 or 1 : 2 ratios). No restrictions were placed on trial location, clinical procedure, or patient severity. We excluded pediatric patients, patients who did not undergo induction of anesthesia, or patients who did not undergo airway manipulation. We also excluded observational studies, review articles, erratum, letters, and notes.

### 2.2. Data Sources and Search Strategies

A comprehensive search of several databases was conducted by a medical reference librarian. The databases included Ovid MEDLINE, Epub Ahead of Print, Ovid Medline In-Process and Other Non-Indexed Citations, Ovid MEDLINE, Ovid EMBASE, Ovid Cochrane Central Register of Controlled Trials, Ovid Cochrane Database of Systematic Reviews, and Scopus. Controlled vocabulary supplemented with keywords was used to search for the trials. We limited our search from January 1, 2000, to October 17, 2018. The search strategy is listed in Table 1.

### 2.3. Trial Selection

Reviewers, working independently, screened abstracts and titles for eligibility using the above inclusion and exclusion criteria. Full-text articles were then further screened using the same criteria. At the level of full-text screening, any disagreements were resolved by consensus between the two reviewers (MS and AM) or by consulting a third reviewer (NJS).

### 2.4. Outcomes

The primary outcomes were hemodynamics (heart rate, systolic and diastolic blood pressure, and MAP) at 5 and 10 minutes following intravenous anesthetic administration. The secondary outcome of interest was pain score as assessed by the visual analog scale (VAS) during the 24 hours postdrug administration.

### 2.5. Methodological Quality and Certainty of Evidence

The risk of bias was assessed using the Cochrane Collaboration's tool for randomized clinical trials. We assessed random sequence generation, allocation concealment, blinding of providers, outcome assessors, and patients, incomplete outcome data, selective outcome reporting (based on the availability of protocol and inclusion of all prespecified outcomes), and other sources of bias (conflict of interest, source funding, risk of bias due to deviations from the intended interventions, etc.). Disagreements were resolved by consensus between two reviewers (MS and AM). The overall certainty across trials for each outcome was appraised by discussion between the two reviewers using the grading of recommendations assessment, development, and evaluation (GRADE) approach. Using the grading of recommendations assessment, development, and evaluation approach, randomized trials, as in this systematic review, would provide a starting level of certainty that is high. This level can be downgraded based on the risk of bias of the individual trials, inconsistency in the results, indirectness, imprecision, and other considerations to provide a global assessment of the certainty warranted by the body of evidence [23].

### 2.6. Data Extraction

Two reviewers (MS and AM) independently extracted the following information from each trial: author, publication year, patient characteristics, intervention, comparison, and outcomes.

### 2.7. Data Synthesis and Statistical Analysis

We extracted or calculated the weighted mean difference (WMD) for continuous outcomes and relative risk for binary outcomes with associated 95% confidence intervals (CIs) from the included studies. Outcomes without measures of variations (e.g., standard deviation, standard error, and CI) were not included in the meta-analysis. The DerSimonian and Laird random-effects models were used to generate combined effects [24]. A two-sided *P* value of less than or equal to 0.05 was used to determine significance for the secondary outcome. To evaluate heterogeneity, we calculated the *I*^2^ statistic [25, 26] and heterogeneity *P* values, where *I*^2^ more than 50% or a *P* value of less than 0.05 suggests high heterogeneity. All statistical analyses were performed using Stata, version 15.0 (StataCorp 2017, College Station, Texas, United States).

## 3. Results

### 3.1. Trial Inclusion

**Figure 1:**
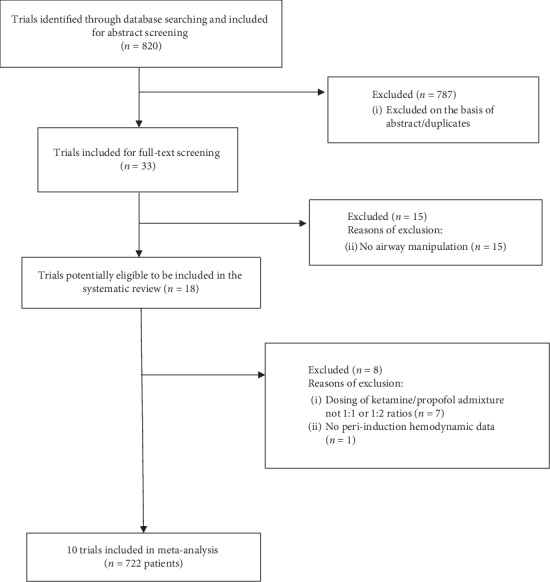
Trial selection.

The searches identified 820 trials. After excluding the irrelevant trials, 33 full-text articles were assessed for eligibility, of which 10 randomized controlled trials (30%) met all criteria and were included in data analysis (*n* = 722) (Figure 1). All the trials were reported in full-length journal articles.

### 3.2. Trial Characteristics

There were a total of 10 trials with 722 patients included. The number of patients in each trial ranged from 40 to 100 (mean, 72.22) with a mean age of 53.99 years. The proportion of participants who were female ranged from 0 to 100% (mean, 39.96%). The American Society of Anesthesiologists physiologic status among participants ranged from I to III. Publication dates ranged from 2000 to 2018 (median, 2011 (all trials published after 2000)). The included trials were conducted in several different countries (four trials in Turkey, three trials in Iran, and one trial from India, Japan, Egypt, and United States). The characteristics of the included trials with dosing are summarized in Table 2.

### 3.3. Risk of Bias

Overall, the risk of bias of included trials was high due to inadequate allocation concealment (7/10, 70%), partial blinding of outcome assessor (4/10, 40%), and selective reporting (9/10, 90%). The risk of summary bias is provided in Table 3.

### 3.4. Heart Rate

**Figure 2:**
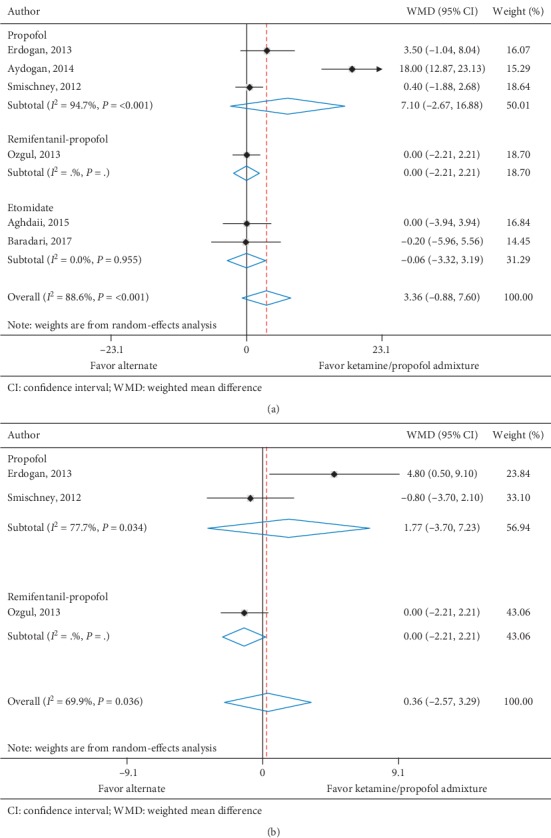
(a) Heart rate at 5 minutes. (b) Heart rate at 10 minutes.

Heart rate at 5 minutes was reported in six trials (*n* = 465) [11, 28–31, 34], and three trials (*n* = 244) [11, 31, 34] reported heart rate at 10 minutes. All trials assessed ketamine/propofol admixture, four compared to propofol and two compared to etomidate at 5 minutes and three compared to propofol at 10 minutes. Patients in the ketamine/propofol admixture group had a nonsignificant increase in heart rate at 5 minutes (WMD, 3.36 beats per minute (95% CI, −0.88 to 7.60), *I*^2^ = 88.6%) and 10 minutes (WMD, 0.36 beats per minute (95% CI, −2.57 to 3.29), *I*^2^ = 69.9%) compared to patients in the nonketamine/propofol admixture group (Table 4 and Figures 2(a) and 2(b)).

### 3.5. Systolic Blood Pressure

**Figure 3:**
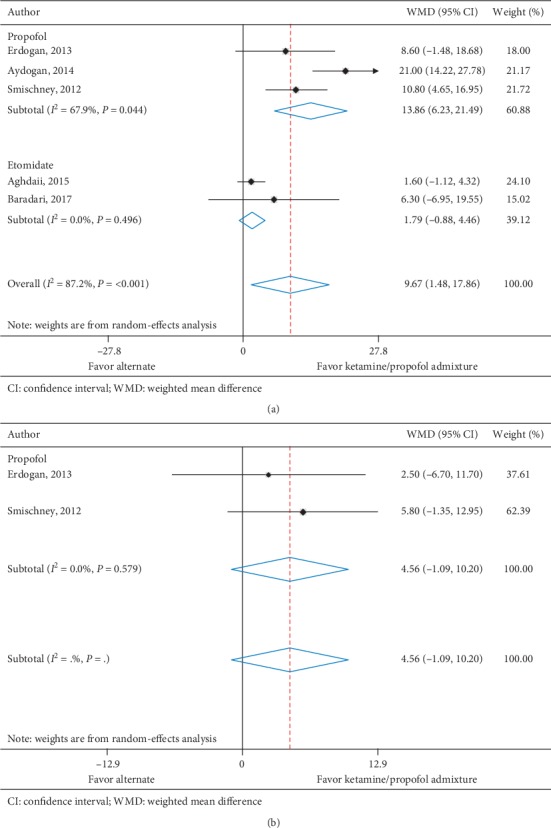
(a) Systolic blood pressure at 5 minutes. (b) Systolic blood pressure at 10 minutes.

Systolic blood pressure at 5 minutes was reported in five trials (*n* = 385) [11, 28–31] and two trials (*n* = 164) [11, 31] reported systolic blood pressure at 10 minutes. All trials assessed ketamine/propofol admixture, three compared to propofol and two compared to etomidate at 5 minutes and two compared to propofol at 10 minutes. Ketamine/propofol admixture was associated with a statistically significant increase in systolic blood pressure at 5 minutes when compared to nonketamine/propofol admixture intravenous anesthetics (WMD, 9.67 mmHg (95% CI, 1.48 to 17.86), *I*^2^ = 87.2%). At 10 minutes, patients in the ketamine/propofol admixture group had a nonsignificant increase in systolic blood pressure compared to patients in the nonketamine/propofol admixture group (WMD, 4.56 mmHg (95% CI, −1.09 to 10.20), *I*^2^ = 0.0%) (Table 4 and Figures 3(a) and 3(b)).

### 3.6. Diastolic Blood Pressure

**Figure 4:**
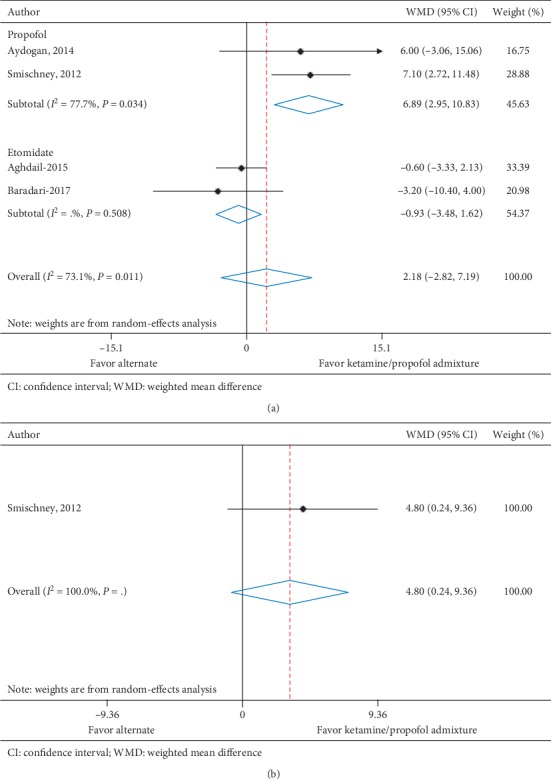
(a) Diastolic blood pressure at 5 minutes. (b) Diastolic blood pressure at 10 minutes.

Diastolic blood pressure at 5 minutes was reported in four trials (*n* = 305) [11, 28–30] and one trial (*n* = 84) [11] reported diastolic blood pressure at 10 minutes. All trials assessed ketamine/propofol admixture, two compared to propofol and two compared to etomidate at 5 minutes and one trial compared to propofol at 10 minutes. Patients in the ketamine/propofol admixture group had a nonsignificant increase in diastolic blood pressure at 5 minutes (WMD, 2.18 mmHg (95% CI, −2.82 to 7.19), *I*^2^ = 73.1%) compared to patients in the nonketamine/propofol admixture group. At 10 minutes, there was a statistically significant increase in diastolic blood pressure in the ketamine/propofol admixture group compared to the nonketamine/propofol admixture group (WMD, 4.80 mmHg (95% CI, 0.24 to 9.36), *I*^2^ = not applicable) (Table 4 and Figures 4(a) and 4(b)).

### 3.7. Mean Arterial Pressure

**Figure 5:**
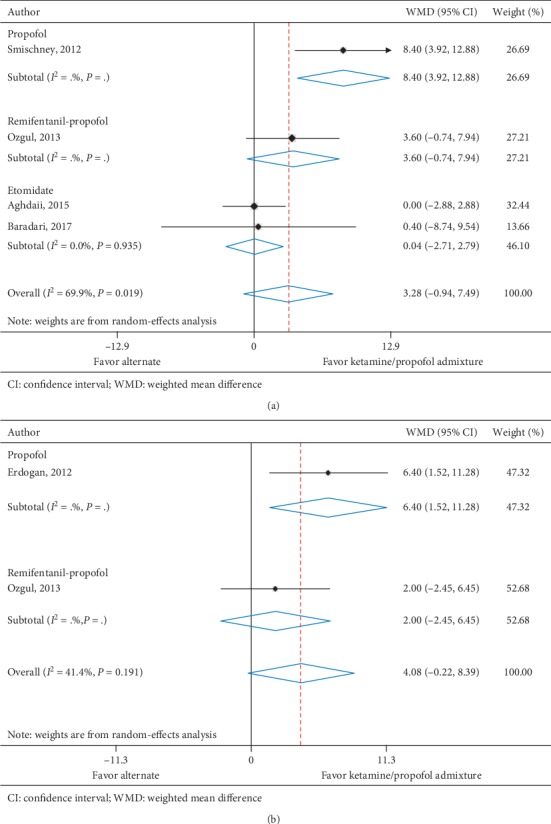
(a) Mean arterial pressure at 5 minutes. (b) Mean arterial pressure at 10 minutes.

Mean arterial pressure at 5 minutes was reported in four trials (*n* = 345) [11, 28, 30, 34] and 2 trials (*n* = 164) [11, 34] reported MAP at 10 minutes. All trials assessed ketamine/propofol admixture, two compared to propofol and two compared to etomidate at 5 minutes and two compared to propofol at 10 minutes. Patients in the ketamine/propofol admixture group had a nonsignificant increase in MAP at 5 minutes (WMD, 3.28 mmHg (95% CI, −0.94 to 7.49), *I*^2^ = 69.9%) and 10 minutes (WMD, 4.08 mmHg (95% CI, −0.22 to 8.39), *I*^2^ = 41.4%) compared to patients in the nonketamine/propofol admixture group (Table 4 and Figures 5(a) and 5(b)).

### 3.8. Pain

Only one trial assessed postprocedural pain by VAS (*n* = 60) [27]. Twenty-six patients in the propofol plus dexmedetomidine group and all patients in the ketamine/propofol admixture group (30 patients) had VAS 1-2 (difference between groups, *P*=0.12). Four patients in the propofol plus dexmedetomidine group had higher pain scores (VAS 3–5) whereas no patients in the ketamine/propofol admixture group had high pain scores (VAS 3–5, difference between groups, *P* ≤ 0.05).

## 4. Discussion

The present systematic review and meta-analysis demonstrate that ketamine/propofol admixture results in a potentially better hemodynamic profile as compared to other agents used for induction of anesthesia. In particular, systolic blood pressure was significantly higher with ketamine/propofol admixture sedation as compared to nonketamine/propofol admixture sedation when induction of anesthesia is required for airway manipulation. Although not statistically significant, heart rate, diastolic blood pressure, and MAP were higher when ketamine/propofol admixture-based sedation was employed versus nonketamine/propofol admixture based-sedation for induction of anesthesia. Furthermore, one study indicated a possible beneficial effect on pain scores in the immediate 24 hours postdrug administration [27].

Evidence has demonstrated that maintaining hemodynamics around the time of airway instrumentation such as endotracheal intubation is vitally important, in both the critically ill and noncritically ill. For example, in the critically ill, peri-intubation hypotension was associated with increased odds of dying (39% peri-intubation hypotension versus 30% no peri-intubation hypotension, *P*=0.045) and increased odds of experiencing intensive care unit length of stay greater than 14 days, duration of mechanical ventilation longer than 7 days, and requiring renal replacement therapy (odds ratio 2.0, 95% CI: 1.30–3.07, *P*=0.0017) [1]. These observations have been demonstrated in other populations of critically ill patients [36]. In noncritically ill patients undergoing noncardiac surgery, a significant fraction of all hypotensive events occurred before skin incision and, therefore, due to anesthetic management as demonstrated in one study. In addition, this study revealed that the odds of developing acute kidney injury increased with MAP less than 65 mmHg (odds ratio 1.02, 95% CI: 1.01–1.04, *P*=0.004) [37]. Moreover, patients undergoing noncardiac surgery who experienced intraoperative hypotension defined as MAP less than 65 mmHg or a MAP decrease of 20% from baseline had increased odds of developing both myocardial and kidney injury [38]. Likewise, patients who developed greater than a 30% decrease in MAP from baseline intraoperatively had increased odds of developing postoperative stroke (odds ratio 1.013/min hypotension, 99.9% CI: 1.000–1.025, *P* ≤ 0.001) [39].

A recent systematic review suggested poor outcomes with increased end-organ injury in patients undergoing noncardiac surgery who experience MAP decreases <80 mm Hg for ≥10 minutes [40]. By the same token, elevated MAP thresholds have been postulated to be beneficial in the critically ill population. For example, one study demonstrated improved microcirculation in septic shock patients with previous hypertension when MAP was increased above a threshold of 65 mmHg [41]. Furthermore, others have shown that maintaining MAP well above 65 mmHg (i.e., 85 mmHg) in septic, critically ill patients may result in less myocardial and kidney injury as well as decreasing overall mortality [42].

Given that periprocedure (i.e., endotracheal intubation) hemodynamics are influenced by sedation and the preponderance of evidence demonstrating that preventing hypotensive episodes, especially with MAPs less than 65 mmHg or SBPs less than 90 mmHg, and maintaining perfusion pressure near baseline improves outcomes, the selection of an intravenous anesthetic agent during a procedure (i.e., endotracheal intubation) ought to have this goal in mind. To this end, a recent editorial questioned the current blood pressure parameter of MAP 65 mmHg in the intensive care unit, which largely comes from two retrospective studies [43]. The authors suggested that perhaps mean perfusion pressure, which is defined by MAP minus central venous pressure, may be a better parameter to use as compared to the current parameter of MAP. While we did not look at long-term outcomes in our current study, we demonstrated that induction with ketamine/propofol admixture rather than other intravenous anesthetic agents resulted in better hemodynamics. Therefore, it is possible that this may translate into improved patient-centered outcomes. Although elevated perfusion pressures may be advantageous in the general sense, this has to be individually tailored to each patient as high pressure/heart rate may be harmful to some. For example, ketamine may exacerbate myocardial ischemia in noncompensated coronary artery disease or hypertension through its effects on central nervous system stimulation and inhibition of norepinephrine reuptake (i.e., cardiac population) [10].

The current systematic review and meta-analysis are consistent with prior reviews of ketamine/propofol admixture administration for procedural sedation. Prior reviews have demonstrated better hemodynamics with ketamine/propofol admixture-based procedural sedation than with nonketamine/propofol admixture-based procedural sedation. For example, a recent review demonstrated that ketamine/propofol admixture-based procedural sedation was effective in reducing cardiovascular complications (relative risk for hypotension 0.11, 95% CI: 0.17–0.97, *P*=0.04; relative risk for bradycardia 0.47, 95% CI: 0.28–0.72, *P*=0.008). The authors also demonstrated similar rates of psychotomimetic complications and nausea-vomiting when compared to propofol [18]. A second review assessed adverse respiratory events and recovery times with ketamine/propofol admixture-based procedural sedation as compared to propofol-based procedural sedation. The authors noted that adverse respiratory events were significantly reduced with ketamine/propofol admixture as compared to propofol (risk ratio 0.82, 95% CI: 0.68–0.99, *P*=0.04); however, recovery times were similar in both groups. Hemodynamic thresholds were not assessed in this review [44]. Although the above reviews point towards the safety of this combination, the admixture can, by its very nature, lead to some undesired effects. Apart from the use of ketamine in the cardiac population (discussed above), ketamine causes initial release of glutamate through nicotinamide adenine dinucleotide phosphate hydrogen oxidase 2 leading to emergence delirium with eventual blockade of glutamate's effects through N-methyl-D-aspartate antagonism. Emergence delirium could be devastating to patients with underlying mental illnesses such as schizophrenia, bipolar disorder, or posttraumatic stress disorder to name a few. This emergence delirium is dose dependent and also affected by cointerventions such as benzodiazepine administration. Interestingly, propofol has been shown to blunt glutamate's effects through gamma-aminobutyric acid_A_ agonist activity [10]. This may explain the findings in the literature demonstrating a reduced rate of emergence delirium with the admixture (∼30% with ketamine only vs. <5%) [10–12, 14, 31].

Our study has several benefits. First, we focused on a theoretical advantage of ketamine/propofol admixture over other agents used for induction of anesthesia during procedures such as endotracheal intubation, namely, hemodynamic preservation. Furthermore, we extracted hemodynamic data in the immediate period after drug administration, thereby limiting the effects of other cointerventions. Second, we extracted data on all drug comparisons to ketamine/propofol admixture. Third, we included trials that had either similar or near equivalent dosing of ketamine/propofol admixture.

### 4.1. Limitations

Similar to other systematic reviews and meta-analyses, our results are limited by clinical trial quality. Although our search strategy was comprehensive, we included only English-language articles and published articles after 2000 and thus may have missed trials not published in English or published before the year 2000. Hemodynamic data were not recorded similarly in all trials nor was the frequency of hemodynamic data collected. Furthermore, 5 and 10 minutes may be an oversimplification of hemodynamic representation as intravenous anesthetics used for induction of anesthesia have a short initial elimination half-life and, therefore, collecting hemodynamic data every minute would have been ideal [10]. Moreover, we did not calculate MAP from trials reporting systolic and diastolic blood pressure because individual patient data were not reported. Three studies met our inclusion criteria and were included in the systematic review. However, we did not include these studies in the meta-analysis as we were unable to extract measures of variation [32, 33, 35]. Data on vasoactive medications which could alter hemodynamics and affect our estimates were not reported in the majority of included trials. We do not report subgroup meta-analyses for the different patient populations or interventions studied due to the limited number of studies in each meta-analysis (only 1 meta-analysis included 3 studies and the remainder had 2 studies). Thus, any subgroup and sensitivity analyses would reduce the number further with only 1 study, which is already presented well in the forest plots. Lastly, our systematic review illustrated moderate to high heterogeneity in the analyses and high risk of bias of the included studies. With such small numbers of studies in the meta-analysis, advanced bias evaluations were not appropriate. Thus, our results should be interpreted with caution.

## 5. Conclusions

We demonstrated a potentially better hemodynamic profile with ketamine/propofol admixture-based induction of anesthesia versus nonketamine/propofol admixture-based induction of anesthesia for procedures requiring airway manipulation such as endotracheal intubation. However, given moderate to high heterogeneity of trials included in the current meta-analysis, our results should be interpreted with caution.

## Figures and Tables

**Table 1:** Database search strategy.

#	Searches	Results
1	exp Propofol/	66561
2	exp Ketamine/	42453
3	1 and 2	6807
4	exp Anesthetics, combined/	232731
5	exp Drug Therapy, Combination/	496978
6	3 and (4 or 5)	5984
7	((ketamine adj2 propofol) or ketofol).ti,ab,hw,kw.	2372
8	From 6 keep 5441–5620	180
9	7 or 8	2519
10	exp evidence based medicine/	1070204
11	exp meta analysis/	242451
12	exp Meta-Analysisas topic/	55758
13	exp “systematic review”/	178680
14	exp controlled study/	6264333
15	exp Randomized Controlled Trial/	971579
16	exp triple blind procedure/	194
17	exp Double-Blind Method/	420309
18	exp Single-Blind Method/	76722
19	exp latin square design/	353
20	exp Placebos/	342744
21	exp Placebo Effect/	10767
22	exp Cohort Studies/	2330769
23	exp retrospective study/	1404531
24	exp prospective study/	1038291
25	((evidence adj based) or (meta adj analys^*∗*^) or (systematic^*∗*^ adj3 review^*∗*^) or (control^*∗*^ adj3 study) or (control^*∗*^ adj3 trial) or (randomized adj3 study) or (randomized adj3 trial) or (randomised adj3 study) or (randomised adj3 trial) or “pragmatic clinical trial” or (random^*∗*^ adj1 allocat^*∗*^) or (doubl^*∗*^ adj blind^*∗*^) or (doubl^*∗*^ adj mask^*∗*^) or (singl^*∗*^ adj blind^*∗*^) or (singl^*∗*^ adj mask^*∗*^) or (tripl^*∗*^ adj blind^*∗*^) or (tripl^*∗*^ adj mask^*∗*^) or (trebl^*∗*^ adj blind^*∗*^) or (trebl^*∗*^ adj mask^*∗*^) or “Latin square” or placebo^*∗*^ or nocebo^*∗*^ or cohort^*∗*^ or retrospectiv^*∗*^ or prospectiv^*∗*^ or (random^*∗*^ and (trial or study))).mp,pt.	12961593
26	or/10–25	13401432
27	9 and 26	1493
28	limit 27 to (editorial or erratum or letter or note or addresses or autobiography or bibliography or biography or blogs or comment or dictionary or directory or interactive tutorial or interview or lectures or legal cases or legislation or news or newspaper article or overall or patient education handout or periodical index or portraits or published erratum or video-audio media or webcasts) (limit not valid in Embase, CCTR,CDSR, Ovid MEDLINE(R), Ovid MEDLINE(R) Daily Update, OvidMEDLINE(R) In-Process, Ovid MEDLINE(R) Publisher; records were retained)	13
29	From 28 keep 11	1
30	(27 not 28) or 29	1481
31	limit 30 to yr = “2000-Current”	1296
32	Remove duplicates from 31	817
Database(s): Embase 1988 to 2018 Week 42, EBM Reviews-Cochrane Central Register of Controlled Trials September 2018, EBM Reviews-Cochrane Database of Systematic Reviews 2005 to October 11, 2018, Ovid MEDLINE(R) and Epub Ahead of Print, In-Process and Other Non-Indexed Citations and Daily 1946 to October 16, 2018		
*Scopus*		
1	TITLE-ABS-KEY((ketamine W/2 propofol) OR ketofol)	
2	TITLE-ABS-KEY((evidence W/1 based) OR (meta W/1 analys^*∗*^) OR (systematic^*∗*^ W/3 review^*∗*^) OR (control^*∗*^ W/3 study) OR (control^*∗*^ W/3 trial) OR (randomized W/3 study) OR (randomized W/3 trial) OR (randomised W/3 study) OR (randomised W/3 trial) OR “pragmatic clinical trial” OR (random^*∗*^ W/1 allocat^*∗*^) OR (doubl^*∗*^ W/1 blind^*∗*^) OR (doubl^*∗*^ W/1 mask^*∗*^) OR (singl^*∗*^ W/1 blind^*∗*^) OR (singl^*∗*^ W/1 mask^*∗*^) OR (tripl^*∗*^ W/1 blind^*∗*^) OR (tripl^*∗*^ W/1 mask^*∗*^) OR (trebl^*∗*^ W/1 blind^*∗*^) OR (trebl^*∗*^ W/1 mask^*∗*^) OR “Latin square” OR placebo^*∗*^ OR nocebo^*∗*^ OR cohort^*∗*^ OR retrospectiv^*∗*^ OR prospectiv^*∗*^ OR (random^*∗*^ and (trial or study)))	
3	PUBYEAR AFT 1999 4 1 and 2 and 3	
5	DOCTYPE(le) OR DOCTYPE(ed) OR DOCTYPE(bk) OR DOCTYPE(er) OR DOCTYPE(no) OR DOCTYPE(sh) 6 4 and not 5	
7	PMID(0^*∗*^) OR PMID(1^*∗*^) OR PMID(2^*∗*^) OR PMID(3^*∗*^) OR PMID(4^*∗*^) OR PMID(5^*∗*^) OR PMID(6^*∗*^) OR PMID(7^*∗*^) OR PMID(8^*∗*^) OR PMID(9^*∗*^) 8 6 and not 7	

**Table 2:** Trial and participant characteristics.

Author, year	Intervention -other adjuncts	Dose description	Patients	Female, (%)	Age, years (mean ± SD)	Weight, kg (mean ± SD) or median (range)	Care location	ASA class (%)
Abdalla, 2015 [27]	Ketamine/propofol admixture *-Atracurium*	Ketamine: 1 mg/kg Propofol: 2 mg/kg	30	46.6	38 ± 10.7	61.8 ± 9.7	Procedural suite	II: 80 III: 20
	Dexmedetomidine + propofol *-Atracurium*	Dexmedetomidine: 1 mcg/kg Propofol: 2 mg/kg	30	33.3	42.7 ± 8.7	60.7 ± 8.5	Procedural suite	II: 86 III: 14

Aghdaii, 2015 [28]	Ketamine/propofol admixture *-Sufentanil* *-Cisatracurium*	Ketamine: 1 mg/kg Propofol: 1 mg/kg	50	30	57.36 ± 5.5	72.06 ± 8.7	OR	II: 60 III: 40
	Etomidate + midazolam *-Sufentanil* *-Cisatracurium*	Etomidate: 0.2 mg/kg Midazolam: 0.06 mg/kg	50	34	57.16 ± 5.6	71.28 ± 11.2	OR	II: 66 III: 34

Aydogan, 2014 [29]	Ketamine/propofol admixture	Ketamine: 100 mg Propofol: 100 mg	20	55	70 ± 65.83	67 (50–100)	OR	I: 45 II: 55
	Propofol	Propofol: 200 mg	20	50	69 ± 65.83	68 (50–102)	OR	I: 50 II: 50

Baradari, 2017 [30]	Ketamine/propofol admixture *-Fentanyl* *-Midazolam -Atracurium*	Ketamine: 1 mg/kg Propofol: 1.5 mg/kg	41	39.02	58.71 ± 9.2	BMI: 26.85 ± 3.89	OR	I: 35 II: 65
	Etomidate + placebo *-Fentanyl* *-Midazolam* *-Atracurium*	Etomidate: 0.2 mg/kg Placebo: normal saline	40	30	62.23 ± 6.3	BMI: 25.23 ± 4.02	OR	I: 49 II: 51

Erdogan, 2013 [31]	Ketamine/propofol admixture *-Fentanyl*	Ketamine: 0.75 mg/kg Propofol: 0.75 mg/kg	40	0	71.67 ± 7.1	71.05 ± 9.37	OR	I: 30 II: 70
	Propofol *-Fentanyl*	Propofol: 0.15 ml/kg (10 mg/ml)	40	0	70.85 ± 5.95	71.32 ± 9.58	OR	I: 35 II: 65

Hosseinzadeh, 2013 [32]	Ketamine/propofol admixture *-Fentanyl* *-Midazolam* *-Atracurium*	Ketamine: 0.75 mg/kg Propofol: 1 mg/kg	30	46.7	65.97 ± 9.31	73.48 ± 8.98	OR	I: 20 II:73.3 III: 6.7
	Etomidate + propofol *-Fentanyl* *-Midazolam* *-Atracurium*	Etomidate: 0.2 mg/kg Propofol: 1 mg/kg	32	34.4	63.91 ± 10.05	72.00 ± 10.73	OR	I: 12.5 II: 68.8 III: 18.7

Iwata, 2009 [33]	Propofol + placebo *-Fentanyl* *-Atropine* *-Vecuronium*	Propofol: 2.0 mg/kg Placebo: normal saline	15	46.6	68 ± 10	58 ± 11	OR	N/R
	Ketamine/propofol admixture 0.5 *-Fentanyl* *-Atropine* *-Vecuronium*	Ketamine: 0.5 mg/kg Propofol: 2 mg/kg	15	46.6	69 ± 5	56 ± 10	OR	N/R
	Ketamine/propofol admixture 1.0 *-Fentanyl* *-Atropine* *-Vecuronium*	Ketamine: 1 mg/kg Propofol: 2 mg/kg	15	33.3	66 ± 10	56 ± 10	OR	N/R

Ozgul, 2013 [34]	Ketamine/propofol admixture *-Remifentanil*	Ketamine: 0.2 ml/kg (5 mg/ml) Propofol: 0.2 ml/kg (5 mg/ml)	40	45	37.75 ± 9.6	70.35 ± 12.42	OR	N/R
	Propofol *-Remifentanil*	Propofol: 0.2 ml/kg (10 mg/ml)	40	57.5	41.47 ± 12.86	73.52 ± 12.61	OR	N/R

Smischney, 2012 [11]	Ketamine/propofol admixture *-Fentanyl* *-Midazolam*	Ketamine: 0.75 mg/kg Propofol: 1.5 mg/kg	41	65	42 ± 12	68.9 ± 11.2	OR	I: 35 II: 65
	Propofol *-Fentanyl* *-Midazolam*	Propofol: 2 mg/kg	43	65	43 ± 11	69.2 ± 11.7	OR	I: 49 II: 51

Vora, 2005 [35]	Ketamine/propofol admixture *-Fentanyl* *-Midazolam*	Ketamine: 50 mg Propofol: 100 mg	30	33.3	42 ± 7.05	49.4 ± 11.7	Procedural suite	I-II: 100
	Propofol + thiopentone *-Fentanyl* *-Midazolam*	Propofol: 100 mg Thiopentone: 125 mg	30	43.3	40.05 ± 9.28	53.6 ± 10.4	Procedural suite	
	Propofol + lignocaine *-Fentanyl* *-Midazolam*	Propofol: 190 mg Lignocaine: 20 mg	30	30	39.6 ± 11.9	51 ± 9.52	Procedural suite	

ASA: American Society of Anesthesiologists; BMI: body mass index; kg: kilograms; mcg: micrograms; mg: milligrams; ml: milliliters; N/R: not reported; OR: operating room; SD: standard deviation.

**Table 3:** Risk of bias.

Trial	Random sequence generation	Allocation concealment	Participant/personnel blinding	Blinding of outcome assessment	Incomplete outcome data, % loss to follow-up	Selective reporting	Other sources of bias	Overall risk
Abdalla, 2015	Low risk	Unclear	Low risk	Unclear	Low risk, 0%	Unclear	Unclear	High
Aghdaii, 2015	Low risk	Unclear	Unclear	Low risk	Unclear, N/R	Unclear	Unclear	High
Aydogan, 2014	Low risk	Low risk	Low risk	Low risk	Low risk, 0%	Unclear	Low risk	Low
Baradari, 2017	High risk	Low risk	Low risk	Low risk	Low risk, 3.5%	Low risk	Low risk	Low
Erdogan, 2013	Low risk	Unclear	Low risk	Unclear	Low risk, 0%	Unclear	Unclear	High
Hosseinzadeh, 2013	Low risk	Unclear	Low risk	Low risk	Unclear, N/R	Unclear	Low risk	Moderate
Iwata, 2009	Low risk	Unclear	Unclear	Low risk	Unclear, N/R	Unclear	Unclear	High
Ozgul, 2013	Low risk	Unclear	Low risk	Unclear	Unclear, N/R	Unclear	Unclear	High
Smischney, 2012	Low risk	Low risk	Low risk	Low risk	Low risk, 0%	Unclear	Low risk	Low
Vora, 2005	Unclear	Unclear	Low risk	Unclear	Unclear, N/R	High risk	Unclear	High

N/R: not reported.

**Table 4:** Hemodynamic outcomes.

Intervention	Outcome	Conclusion	Study design/sample size	Certainty of evidence
Ketamine/propofol admixture vs. nonketamine/propofol admixture	HR 5 min	WMD, 3.36 mmHg (95% CI, −0.88 to 7.60), *I*^2^ = 88.6%	Six RCTs (*n* = 465) 11, 27–31	Very low due to risk of bias, imprecision, and inconsistency
	HR 10 min	WMD, 0.36 mmHg (95% CI, −2.57 to 3.29), *I*^2^ = 69.9%	Three RCTs (*n* = 244) 11, 27, 29	Very low due to risk of bias, imprecision, and inconsistency
	SBP 5 min	WMD, 9.67 mmHg (95% CI, 1.48 to 17.86), *I*^2^ = 87.2%	Five RCTs (*n* = 385) 11, 27–28, 30–31	Low due to risk of bias and inconsistency
	SBP 10 min	WMD, 4.56 mmHg (95% CI, −1.09 to 10.20), *I*^2^ = 0.0%	Two RCTs (*n* = 164) 11, 27	Low due to risk of bias and imprecision
	DBP 5 min	WMD, 2.18 mmHg (95% CI, −2.82 to 7.19), *I*^2^ = 73.1%	Four RCTs (*n* = 305) 11, 28, 30–31	Very low due to risk of bias, inconsistency, and imprecision
	DBP 10 min	WMD, 4.80 mmHg (95% CI, 0.24 to 9.36), *I*^2^ = N/A	One RCT (*n* = 84) 11	Low due to risk of bias and imprecision
	MAP 5 min	WMD, 3.28 mmHg (95% CI, −0.94 to 7.49), *I*^2^ = 69.9%	Four RCTs (*n* = 345) 11, 29–31	Very low due to risk of bias, imprecision, and inconsistency
	MAP 10 min	WMD, 4.08 mmHg (95% CI, −0.22 to 8.39), *I*^2^ = 41.4	Two RCTs (*n* = 164) 11, 29	Low due to risk of bias and imprecision

CI: confidence interval; HR: heart rate; WMD: weighted mean difference; RCT: randomized controlled trial; SBP: systolic blood pressure; DBP: diastolic blood pressure; MAP: mean arterial pressure; N/A: not applicable.
